# Super-Selective Partial Splenic Embolization for Hereditary Spherocytosis in Children: A Single-Center Retrospective Study

**DOI:** 10.3389/fsurg.2022.835430

**Published:** 2022-02-25

**Authors:** Rui-jue Wang, Li Xiao, Xi-ming Xu, Ming-man Zhang, Qiang Xiong

**Affiliations:** ^1^Chongqing Key Laboratory of Pediatrics, Department of Hepatobiliary Surgery, Children's Hospital of Chongqing Medical University, National Clinical Research Center for Child Health and Disorders, Ministry of Education Key Laboratory of Child Development and Disorders, Chongqing, China; ^2^Department of Medical Record Statistics, Children's Hospital of Chongqing Medical University, National Clinical Research Center for Child Health and Disorders, Chongqing Higher Institution Engineering Research Center of Children's Medical Big Data Intelligent Application, Ministry of Education Key Laboratory of Child Development and Disorders, Chongqing, China

**Keywords:** hereditary spherocytosis, super-selective partial splenic embolization, children, interventional therapy, surgery therapy

## Abstract

**Background:**

Hereditary spherocytosis (HS) is the most common hemolytic anemia due to erythrocyte membrane defects. Total splenectomy is the most effective treatment for moderate or severe HS. As a conservative alternative, partial splenic embolization (PSE) can preserve part of the spleen's function, thus reducing the risk of overwhelming post-splenectomy infection (OPSI) or sepsis, especially for pediatric patients. However, it is not easy to precisely control the scope of interventional embolization, limiting PSE applications. The present study aims to optimize the PSE procedure on smaller, which is named super-selective PSE (SPSE), to improve the controllability and assess the feasibility and effectiveness of SPSE.

**Results:**

This study was conducted by retrospectively reviewing clinical data from HS patients treated by surgical treatments, which were diagnosed at the children's hospital of Chongqing medical university from January 2015 to December 2019. Patients were divided into two groups according to their treatment preference: SPSE (16 patients) group and total splenectomy (41 patients) group. The mean proportion range of splenic embolism by SPSE was 82.4%, close to the expected value (70–85%). The average hemoglobin value was increased significantly from 6.85 (5.6–8.0) g/dl before SPSE to 12.4 (10.4–13.3) g/dl after SPSE (*p* < 0.001). All children after SPSE suffered mild post-embolization syndrome, such as pain, fever, and vomiting, which could easily be controlled with appropriate supportive therapy.

**Conclusions:**

Super-selective partial splenic embolization is a safe and effective treatment for moderate or severe HS in children. However, with a longer follow-up, more patients further assess the value of SPSE.

## Introduction

Hereditary spherocytosis (HS) is hemolytic anemia of varying severity caused by red blood cell membrane defects. Defects in the erythrocyte membrane and selective destruction of defective erythrocytes by the spleen are two critical factors in the pathophysiological process of HS. The destruction of the red blood cells by the spleen can lead to proliferative enlargement of the spleen, which leads to increased red blood cell accumulation, increased hemolysis, or both, thus creating a vicious cycle (1).

The treatment of HS aims to minimize the complications of chronic hemolysis and anemia. In addition to symptomatic treatment and blood transfusion, current guidelines recommend splenectomy if the patient has severe anemia complications or is transfusion-dependent (1–3). Since the spleen is a lymphoid organ, the overwhelming post-splenectomy infection (OPSI) is the most severe post-operative complication, especially in children. In addition to surgical risks, splenic resection is also prone to severe surgical complications, such as venous thromboembolism (VTE), pulmonary arthritis after splenectomy (4). Patients who have indications for splenectomy but were unwilling to undergo surgery, or who have contraindications to surgery, have been reported to be treated with relatively conservative surgery, such as partial splenectomy, partial splenic embolization (PSE), and partial splenic artery ligation, which exhibited better outcomes and fewer surgical complications due to residual partial splenic immunity (5, 6). However, PSE had not been widely studied and applied in pediatric HS patients due to the poor controllability of the embolic range.

Our study aims to accurately predict the extent of splenic embolism by further improving and optimizing PSE for super-selective cannulation of the splenic artery. The present study reports on the feasibility and efficacy of super-selective partial splenic embolization (SPSE) in a cohort of 16 children with HS.

## Study Patients

This study was conducted by retrospectively reviewing clinical data from 58 children patients with HS diagnosed at the Children's Hospital of Chongqing Medical University from January 2015 to December 2020, and the last follow-up visit was October 2021. The Children' s Hospital approved our study of Chongqing Medical University (No: 2020-288), and informed consent was obtained to review patients' medical records. We excluded one patient with splenectomy because follow-up data were lacking, leaving 57 patients for the study. All eligible patients were assessed according to the BCSH 2011 guidelines and European Hematology Association 2017 guidelines (2, 3), while only patients with moderate, severe HS, or impact daily life were operated on. Grouping was based on the treatment intention, 41 patients had undergone total splenectomy (median age 9, range 2–17 years), and 16 patients had undergone SPSE (median age 7 years, range 5–11 years). Before receiving the surgery, all patients and guardians were provided with two surgical consent forms for splenectomy and SPES surgery. The consent forms consist of detailed explanations on the two surgical treatment options to ensure that each patient and guardian fully understood the advantages and disadvantages of both surgical options before making their decision (for details, see Appendix).

## SPSE Surgery Procedure

We performed super-selective embolization of the middle and lower parts of the spleen using splenic artery trunk angiography to analyze the distribution of splenic segmental arteries. The expected embolism range was 70–85%. The specific surgical procedure was as follows:

First, splenic arteriogram procedure: The patients were placed in the supine position, a 4Fr introducer sheath (RS^*^A40G07SQ, TERUMO) was inserted through the right common femoral artery by using the Seldinger technique, and a 4Fr catheter (RF^*^ZB54110, TERUMO) was passed through the main trunk of the splenic artery for imaging. Based on the visualization of the splenic vessels, adopting splenic segmental arteries in the lower and middle spleen as the target vessel, the arterial embolization, which was confirmed, by contrast, was performed by a 2.7 Fr microcatheter (MC-PE27131, TERUMO) to super-select the splenic segmental arteries.

Secondly, the configuration of embolic agents: Mixing polyvinyl alcohol granules (PVA-500, COOK) 1 g and lodixanol 320 (GE Healthcare Ireland) 5 ml thoroughly, extract 2 ml of suspension and add 0.25 g of triple cephalosporin, then add the appropriate amount of contrast agent to make a total of 15 ml of the embolic agent.

Third, embolization procedure: The embolization area was mainly in the middle and lower spleen, preserving most of the upper spleen and the splenic hilum. At the same time, the degree of embolization was judged by the intraoperative flow velocity of the embolized artery. The degree of embolization was determined according to a slight slowing of the flow as 30–40%, a marked slowing as 50–60%, a peristaltic advance after briefly stopped as 70–80%, a significant regurgitation as 90% and above. The extent of splenic embolism was predicted by the above two aspects. After completing embolization, the degree of embolization was determined again by imaging the main trunk of the splenic artery. If the embolic scope is insufficient, additional embolization operations can be performed until the embolic scope reaches 70–85%. Sterile gauze and pressure were applied to the puncture site for hemostasis, followed by bandaging and braking for 6–8 h. The operation time only requires 30 min maximum. The patency of splenic vein flow was detected by vascular ultrasound 3 days after surgery, and third-generation cephalosporin was applied by intravenous prophylactic in 48 h after surgery.

## Data Collection and Follow-Up

We collected pre- and post-operative examination data of patients who underwent total splenectomy and SPSE, with a minimum 12-months follow-up. The last follow-up was in September 2020. The patients underwent CT scan using a 64-channel multidetector CT system (Lig_x0002_htspeed VCT, GE Medical Systems, USA). The Volume Rendering function analyzed splenic volumes with the Philips intellispace portal software platform, version 6.0.1.20700.

## Statistical Methods

Statistical analyses were performed using the SPSS software version 22.0. Measurement data were expressed as mean ± SD and analyzed by Student's *t*-test. Enumeration data were expressed as a rate (%), and a Chi-square test was adopted. Comparison of laboratory parameters after splenic artery embolization was carried out using the paired *t*-test, the Wilcoxon signed ranks test, the Mann–Whitney *U*-test, or the chi-square test. Statistical significance was defined as *p* < 0.05.

## Results

No cases of serious post-operative complications or post-splenectomy infections were found in either group within 6 months after surgery during the follow-up period. There was no statistical difference in the pre-operative condition of the two groups (Table 1), and hemolysis was significantly improved in both groups. The average hemoglobin value was increased significantly from 7.4 g/dl before total splenectomy to 11.7 g/dl after total splenectomy (*p* < 0.001). The average hemoglobin value was increased significantly from 6.97 g/dl before SPSE to 12.2 g/dl after SPSE (*p* < 0.001).

**Table 1:** Comparison of clinical characteristics of patients undergoing total splenectomy and super-selective partial splenic embolization (SPSE).

	**total splenectomy**		**SPSE**		**chi-square value /Z value**	***P***
	***N* = 41**	**median (range)**	***N* = 16**	**median (range)**		
male (%)		53.7%		37.5%	1.202	0.273
Mean age at surgery, y		9 (2–17)		7 (5–11)	−1.563	0.118
Mean hemoglobin before surgery, g/L		74 (18–95)		68.5 (56–80)	−0.275	0.783
Mean hemoglobin after surgery, g/L		117 (97–175)		124 (104–133)	−0.703	0.482
Mean free bilirubin before surgery, μmol/L		109.45 (21.80–237.80)		61.4 (16.0–146.0)	−0.071	0.943
Mean free bilirubin after surgery, μmol/L		42.87 (2.80–66.80)		13.35 (7.00–86.00)	−1.633	0.102
Mean platelet count before surgery, × 10^9^/L		188 (27–364)		121 (77–333)	−1.368	0.171
Mean platelet count after surgery, × 10^9^/L		418 (165–1416)		442 (311–643)	−2.246	0.025
Mean white blood cell count before surgery, × 10^9^/L		5.57 (3.28–25.06)		7.37 (5.27–18.00)	−0.906	0.365
Mean white blood cell count after surgery, × 10^9^/L		7.09 (3.68–13.12)		10.11 (3.40–15.70)	−2.790	0.005

**Figure 1:**
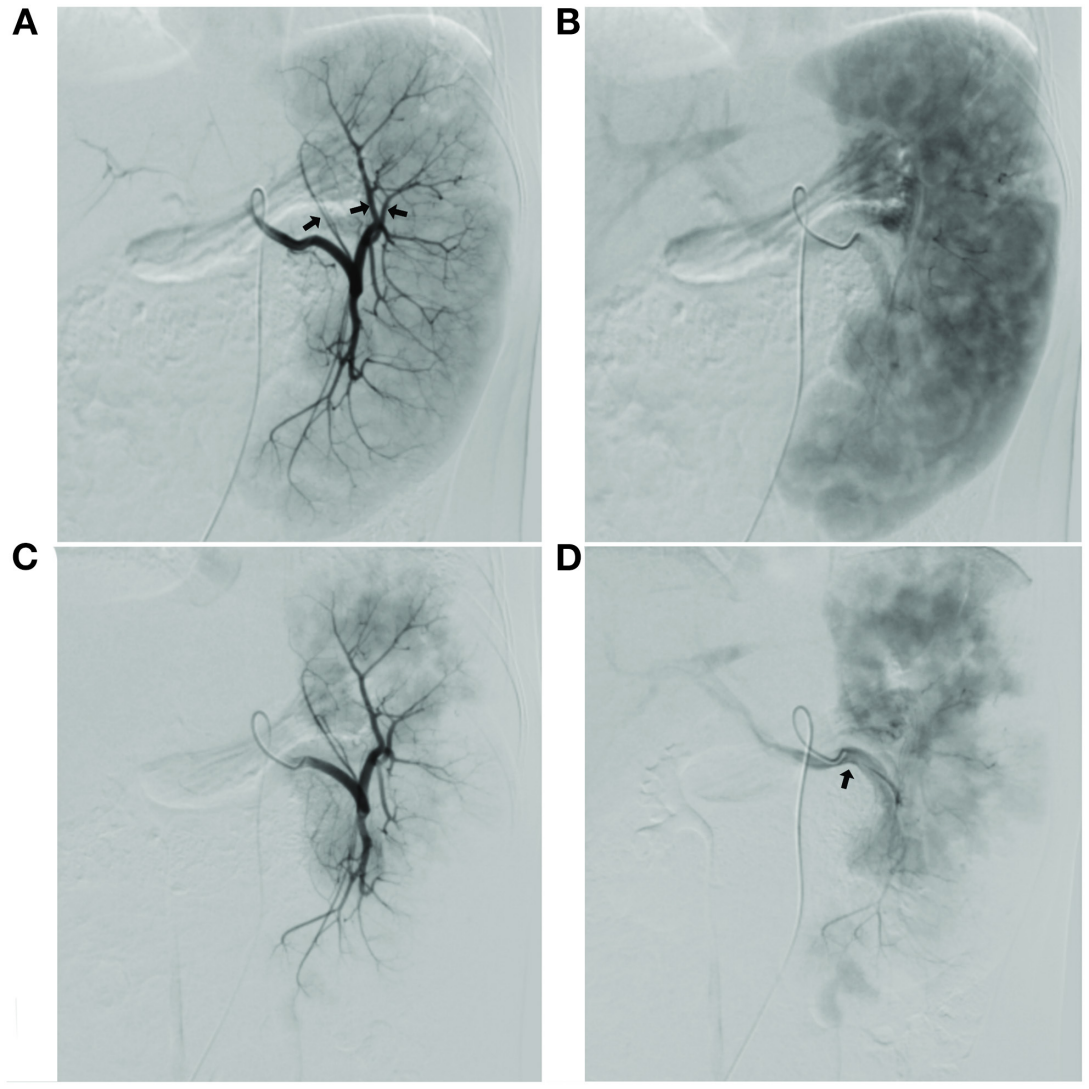
**(A)** Image of the splenic segment arteries in arterial phase, with the planned preservation of the superior pole splenic segment artery shown by the arrow; **(B)** Image of the spleen in the equilibrium phase, showing the full projected area of the spleen; **(C)** Image of splenic segment artery after super-selective partial splenic embolization (SPSE) with preserved superior pole splenic segment artery; image of the spleen in equilibrium after SPSE with the area of residual spleen projection, with estimated embolization extent of up to 90% compared to **(B)**; **(D)** Splenic vein imaging.

The surgical results are in Table 2. Before and after embolization, the arterial phase showed the branches and distribution of the splenic arteries, and the equilibrium phase showed the distribution of the parenchymal blood supply to the spleen (Figure 1).

**Table 2:** General conditions and surgical results in children with partial splenic artery embolization.

**NO**	**Age at diagnosis (y)**	**Age at surgery, (y)**	**Estimated embolism range (%)**	**preoperative spleen volume (cm^**3**^)**	**Postoperative spleen volume^**a**^ (cm^**3**^)**	**Actual embolism range (%)**	**Duration of postoperative abdominal pain (d)**	**Duration of postoperative fever (d)**	**Number of postoperative vomiting**
1	1	5	75	NA	NA	NA	7	6	0
2	4	5	85	NA	NA	NA	6	5	1
3	1	6	80	NA	NA	NA	8	0	0
4	1	7	75	NA	NA	NA	9	0	0
5	2	7	85	NA	NA	NA	6	0	0
6	6	11	85	254.9	30.0	88.2	5	0	1
7	1	7	75	534.0	98.5	81.6	3	0	3
8	6	7	80	545.3	135.0	75.2	7	0	0
9	4	6	75	292.8	78.5	73.2	0	0	0
10	2	7	90	826.8	90.3	89.1	6	0	0
11	5	6	80	263.9	60.9	76.9	5	2	0
12	10	11	90	679.0	112.2	83.5	8	0	2
13	3	8	80	719.5	88.1	87.8	3	0	0
14	3	9	85	598.6	153.9	74.3	6	0	0
15	7	8	85	346.1	48.5	86.0	0	0	1
16	1	5	80	651.2	190.7	70.7	3	1	0

a *CT scan was arranged 6–9 days after SPSE*.

**Figure 2:**
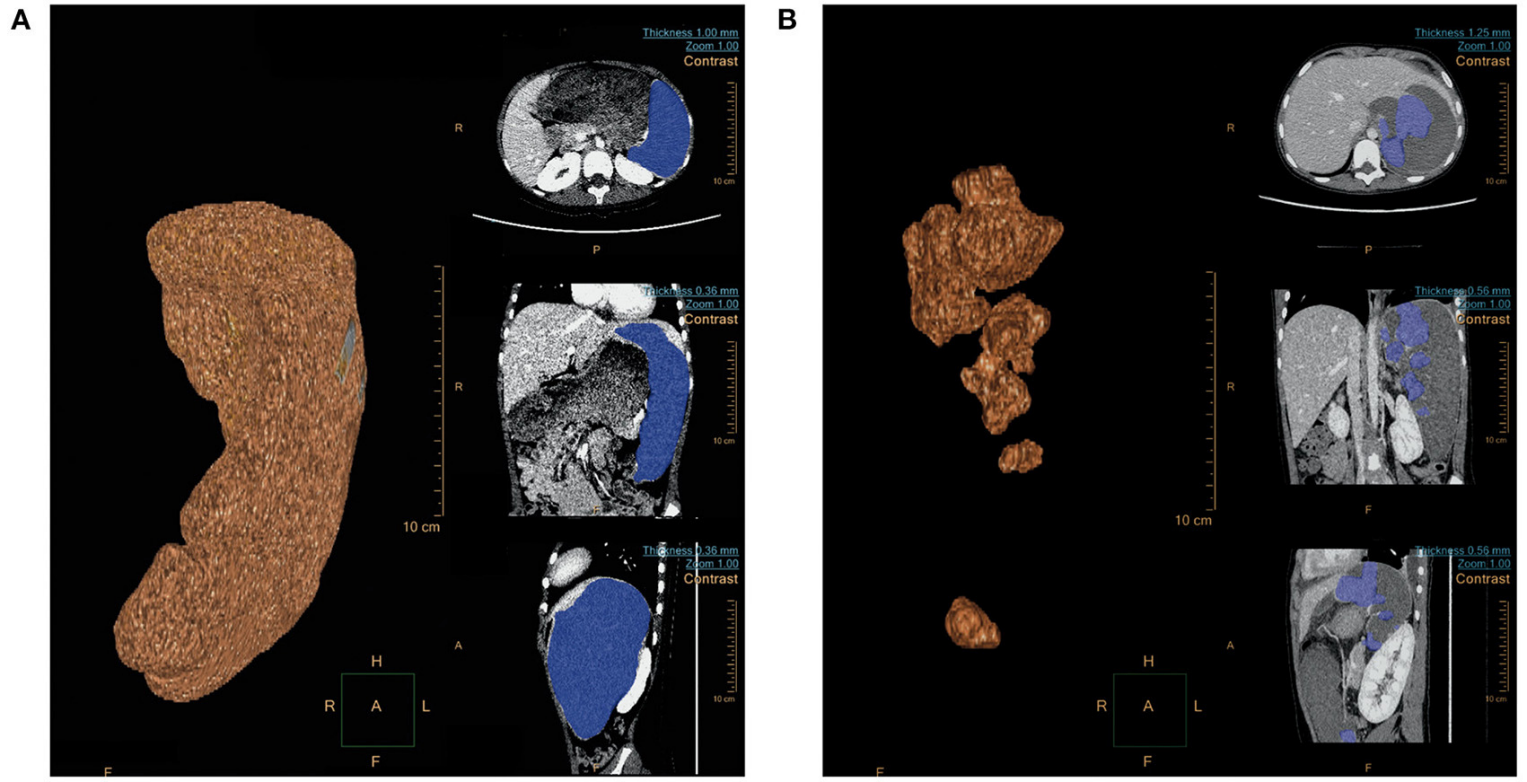
**(A)** Spleen volume (679.0 cm^3^) before embolization; **(B)** Spleen volume (112.2 cm^3^) 1 week after embolization.

The 16 children underwent embolization of the middle and lower pole of the spleen, and the expected intraoperative embolization extent was 75–90%. Pre-embolization and post-embolization (7–9 days) CT examinations were completed in 11 patients. The pre- and post-embolization spleen volumes were calculated, and the actual splenic embolic extent was 73.2–89.1% (Figure 2). The difference between the pre-assessed embolic extent and the actual post-operative embolic extent was not statistically significant. This result suggests that SPSE can accurately determine the extent of splenic embolization intraoperatively.

All patients treated with SPSE had no serious complications (Table 2) but experienced a milder post-embolization syndrome. Approximately 87.5% (14/16) of children presented with mild abdominal pain with median abdominal pain duration of 6 days (range 0–9 days), which could easily be controlled with appropriate supportive therapy. Post-operative fever and vomiting occurred in 25.0% (4/16) and 31.3% (5/16) of the children, both of which resolved rapidly on autonomy. No other serious complications, such as splenic abscess, portal vein embolism, pancreatitis, and pulmonary atelectasis, occurred.

The median follow-up time for this group of patients was 26 months (19–41 months), all children showed significant improvement in hemolysis, and no patient underwent transfusion for hemolysis during the follow-up period. Notably, there were no significant abnormalities in the common immune indicators before and after surgery, suggesting that the patient retained splenic immune function after surgery (Table 3).

**Table 3:** Comparison of clinical indicators before and after surgery in patients undergoing SPSE.

	**preoperative**	**Postoperative ^**a**^**	**t/Z**	***P***
Spleen volume (cm^3^)	545.72 ± 190.97	105.66 ± 45	7.90	<0.001
white blood cell count before surgery, × 10^9^/L	8.38 ± 3.05	9.78 ± 1.91	−1.59	0.132
hemoglobin before surgery	69.73 ± 6.88	122.13 ± 9.86	−16.19	<0.001
platelet count, × 10^9^/L	220.19 ± 60.90	425.20 ± 70.72	−11.64	<0.001
free bilirubin, μmol/L	61.40 (16–146)	13.35 (7.00–86.00)	−3.52	<0.001
IgG, g/L	11.51 ± 2.81	12.21 ± 3.84	−0.99	0.336
IgA, g/L	1.97 ± 0.81	2.17 ± 0.83	−1.30	0.214
IgE, g/L	10.3 (0.6–152)	12.55 (2.6–202)	−1.24	0.211
IgM, g/L	1.32 ± 0.53	1.28 ± 0.52	0.38	0.711
Complement C3, g/L	0.57 ± 0.08	0.82 ± 0.21	−5.90	<0.001
Complement C4, g/L	0.15 ± 0.04	0.20 ± 0.05	−4.75	<0.001

a *6 months after SPSE*.

## Discussion

Total splenectomy is a proven and effective method for treating patients with moderate to severe HS (3). The spleen is the largest peripheral lymphoid organ in the body, and it is an indispensable immune barrier when the body receives external viruses and bacteria. The absence of a spleen might lead to an infection outbreak. For pediatric patients, possible post-operative aggressive complications, such as OPSI and surgical trauma, have been a long-term problem for physicians and patients. In 1973, Maddison (7) proposed splenic artery embolization as a minimally invasive procedure for treating hypersplenism that solves the problem of severe trauma. Spigos et al. (8) developed transcatheter PSE in adult HS to preserve the immune function of the spleen (5, 6). However, PSE has not been widely promoted in pediatric patients because of the poor controllability of the extent of PSE embolization. Later, conservative surgical options (such as partial splenectomy and partial splenic artery ligation) were considered viable options for HS because they preserved sufficient splenic tissue and relieving anemia, but neither could avoid the trauma of laparotomy.

In our study, based on the unique anatomy of the spleen and splenic artery, selective embolization of splenic segmental arteries branches was performed in the spleen by intraoperative angiography. We measured the embolic artery blood flow velocity and performed a comprehensive re-evaluation of the extent of splenic embolization to predict splenic embolic volume accurately. The extent of spleen volume examined by CT before and after embolization was 70–85%, consistent with the pre-evaluation results. Therefore, we believe SPSE can precisely control the degree of splenic embolization to achieve a therapeutic effect of reducing hemolysis. In this study, all the patients had moderate or severe HS. Post-operative hemolysis was significantly improved; no further transfusions were performed in our patients. Previous studies had shown that PSE effectively reduced the degree of hemolysis while maintaining the phagocytic function of the splenic remnant and the selective destruction of the spleen to defective red blood cells (5).

As with partial splenic artery resection and partial splenic artery ligation, this study was also effective in reducing the volume of the spleen and maintaining the immune function of the splenic to some extent (9). Compared to the preoperative period, the mild elevation of the complement system was considered an effect possibly related to surgical embolization (10, 11). The complement system exerts innate immune by lysing or killing bacteria, modulating phagocytosis, or participating in antibody-mediated specific immune responses. Our study also showed that all splenic immune functions were maintained in the normal range after SPSE and no surgery-related infection of SPSE during the follow-up period. Thus, we demonstrated that SPSE substantially improved in maintaining normal immune function than total splenectomy. We are aware that the younger the patients with HS diagnosis, the severer the symptoms of hemolytic anemia may have. Considering the risk of post-splenectomy fulminant infection, many patients with total splenectomy were significantly older. We believe SPSE preserves normal immune function while treating HS offers the possibility of surgery at earlier ages, and reduces complications for patients with HS.

These study's post-embolization syndromes are minor and controllable, mainly manifested by abdominal pain, fever, and vomiting. Most patients' symptoms resolve within 1 week after surgery. 87.5% (14/16) of children had mild abdominal pain with a median duration of 6 days (range 0–9 days), which most children tolerated without medication, and children with more severe abdominal pain were given 1–2 oral nonsteroidal antiinflammatory drugs (NSAIDs) daily (e.g., ibuprofen), post-operative fever lasting 1–6 days was observed in 25.0% (4/16) of the children, who also received ibuprofen orally and had a rapid decrease in temperature to normal, without recurrent daily fever and signs of infection. 31.3% (5/16) of the children had post-operative vomiting lasting 1–3 days, 1–2 times daily, and the vomitus was all gastric juice. All SPSE-treated patients did not develop other serious complications, such as splenic abscess, portal vein embolism, pancreatitis, or pulmonary atelectasis, during the follow-up time. All children treated with splenectomy had wound pain lasting 3–5 days, which most children tolerated without medication, and the more severe children were given oral NSAIDs (e.g., ibuprofen) 1–2 times daily for 2–3 days, all tolerating the pain. All children had fasted for 1–3 days after surgery. 7.3% (3/41) of the children had electrolyte disturbance after surgery, corrected with intravenous rehydration, and recovered. 7.3% (3/41) of the children had severe infections after surgery. On the fourth day after surgery, one case had severe pneumonia and respiratory failure. It was treated with a ventilator in ICU, and one case had pneumonia and atelectasis on the right side 5 days after surgery, which was treated with fiberoptic bronchoscopy and improved. One case developed abdominal infection 33 months after surgery and improved after reoperation. A child with splenectomy was found to have partial portal vein thrombosis during follow-up and improved after anticoagulation.

The previous studies have shown that common complications of total splenectomy include postoperative infection, OPSI, surgical bleeding, injury of adjacent organs, and VTE (2, 12, 13). The most severe complications of PSE are abdominal infection and spleen abscess formation, which is deadly for patients in severe cases (14). The cause of spleen abscess formation includes excessive embolization, which leads to liquefied necrosis of a large amount of spleen tissue and secondary bacterial infection. There are two possible sources of bacteria; one of them is interventional contamination. The other is that the enteric-derived bacteria retained in the portal venous system reverse into the spleen since the slow flow speed in the splenic vein after massive embolization of the splenic parenchyma (15). However, no patient had a splenic abscess in this study. The reason to do this study is as follows. Firstly, the number of patients is still small. Secondly, SPSE achieved the purpose of accurate embolization by the accurate selection of secondary arteries and narrowing the possibility of infection caused by stagnation of splenic vein blood flow, which is the result of over embolization of splenic artery trunk. Thirdly, the minified dosage of embolic agent during treatment also indirectly diminishes the risk of contamination. There is no exposure of significant flow stagnation or reflux in the splenic artery angiogram after SPSE had been confirmed by the color ultrasonography of the blood flow of the splenic vein 3 days after the procedure. In addition, triple cephalosporin was intravenously applied within 48 h postoperatively. Since these preventative anti-infection actions were actively performed, the risk of splenic infection was minimized by avoiding possible intraoperative contamination and post-operative entheogenic infection to the greatest extent.

A few reports of the previous study showed the growth of residual accessory spleen after total splenectomy (16), or growth of residual spleen after partial splenectomy trigger recurrence of anemia which requires secondary surgery (17, 18). In a long-term follow-up study of partial splenectomy (19), mild and moderate hemolysis may be a long-term symptom after partial splenectomy, and a small number of patients may be at risk of secondary gallstones and hemolytic anemia. In our observed SPSE patients with a median follow-up time of 22 months, there are no cases of hemolytic anemia which requires transfusion therapy, only a few children have mild hemolysis, and all patients with SPSE recovered well after surgery. We believe that the better results were mainly because the accurate control of embolization preserved only a tiny portion of the upper splenic (10–25%); moreover, the growth of the normal residual spleen was effectively limited by the blockage of the diaphragm and embolized spleen. Since we preserved the splenic artery trunk upfront, there is an opportunity to do another embolization as a re-intervention if symptomatic recurrence of anemia appeared at a later stage. The shortage of this study is the short follow-up period. Hence, long-term follow-up should be applied to clarify the dynamic changes of spleen volume and hemolysis after embolization. We considered modified super selective partial splenic artery embolization as a conservative surgical treatment for posterior globules.

The current study focuses on the safety of SPSE, so the initial case selection tends to be for the traditional splenectomy indication. We suggest splenectomy for children with HS who remain transfusion-dependent or have severe symptoms related to anemia after 1 year of age. If necessary, a total splenectomy can be postponed until after 6 years of age. Our current study follows the willingness of the child's guardian to treat the choice of surgical approach after detailed knowledge of the advantages and disadvantages of both surgical options, so the fact that the children treated with SPES were above 5 years of age is related to the small sample size. SPES is technically feasible for children with HS at 5 years or even younger. We have treated a 1-year-old child with Wiskott–Aldrich syndrome, and after a multidisciplinary assessment that splenectomy may pose a high risk of death, we successfully managed the symptoms and safely treated the child with SPES until he was treated with a hematopoietic stem cell transplant.

In conclusion, SPSE is a reliable, safe, and effective alternative to splenectomy for childhood HS, which resolved the disadvantage of difficulty in estimating the volume of splenic artery embolization and avoided the trauma of partial splenectomy and partial splenic artery ligation. SPSE may have more tremendous advantages on expanding the age range of surgery, reducing the severity of surgical trauma, and minimizing the possibility of infection. However, a more extended follow-up period, a larger sample size, and applying other indications, such as hypersplenism, are mandatory for the further assessment of SPSE.

## Data Availability Statement

The raw data supporting the conclusions of this article will be made available by the authors, without undue reservation.

## Ethics Statement

The studies involving human participants were reviewed and approved by ethical approval for this study was provided by the Institutional Review Board of CHCMU and registered at No: 2020-288. Written informed consent to participate in this study was provided by the participants' legal guardian/next of kin. Written informed consent was obtained from the minor(s)' legal guardian/next of kin for the publication of any potentially identifiable images or data included in this article.

## Author Contributions

QX and M-mZ conceived the study and are the principal investigator. QX and R-jW initially designed the study protocol and wrote the first draft of this manuscript. M-mZ coordinated the doctors and nurses. At the same time, QX, R-jW, and M-mZ mainly provided clinical support and led the surgery. LX, X-mX, and R-jW collected and analyzed the data. R-jW and LX revised this manuscript. All authors contributed to subsequent drafts and approved the final manuscript.

## Funding

This work was supported by the 2022 Research Projects of Chongqing Municipal Health and Family Planning Commission (No. 2022WSJK005).

## Conflict of Interest

The authors declare that the research was conducted in the absence of any commercial or financial relationships that could be construed as a potential conflict of interest.

## Publisher's Note

All claims expressed in this article are solely those of the authors and do not necessarily represent those of their affiliated organizations, or those of the publisher, the editors and the reviewers. Any product that may be evaluated in this article, or claim that may be made by its manufacturer, is not guaranteed or endorsed by the publisher.
